# Voxel-based morphometry analyses of *in vivo* MRI in the aging mouse lemur primate

**DOI:** 10.3389/fnagi.2014.00082

**Published:** 2014-05-06

**Authors:** Stephen J. Sawiak, Jean-Luc Picq, Marc Dhenain

**Affiliations:** ^1^Wolfson Brain Imaging Centre, University of Cambridge, Addenbrooke's HospitalCambridge, UK; ^2^Behavioural and Clinical Neuroscience Institute, University of CambridgeCambridge, UK; ^3^EA, 2027: Laboratoire de Psychopathologie et de Neuropsychologie, Université Paris 8St-Denis, France; ^4^CEA, DSV, I2BM, MIRCenFontenay-aux-Roses, France; ^5^CNRS, URA 2210Fontenay-aux-Roses, France

**Keywords:** aging, atrophy, mouse lemur, *Microcebus murinus*, MRI, primate, SPMMouse, VBM

## Abstract

Cerebral atrophy is one of the most widely brain alterations associated to aging. A clear relationship has been established between age-associated cognitive impairments and cerebral atrophy. The mouse lemur (*Microcebus murinus*) is a small primate used as a model of age-related neurodegenerative processes. It is the first non-human primate in which cerebral atrophy has been correlated with cognitive deficits. Previous studies of cerebral atrophy in this model were based on time consuming manual delineation or measurement of selected brain regions from magnetic resonance images (MRI). These measures could not be used to analyse regions that cannot be easily outlined such as the nucleus basalis of Meynert or the subiculum. In humans, morphometric assessment of structural changes with age is generally performed with automated procedures such as voxel-based morphometry (VBM). The objective of our work was to perform user-independent assessment of age-related morphological changes in the whole brain of large mouse lemur populations thanks to VBM. The study was based on the SPMMouse toolbox of SPM 8 and involved thirty mouse lemurs aged from 1.9 to 11.3 years. The automatic method revealed for the first time atrophy in regions where manual delineation is prohibitive (nucleus basalis of Meynert, subiculum, prepiriform cortex, Brodmann areas 13–16, hypothalamus, putamen, thalamus, corpus callosum). Some of these regions are described as particularly sensitive to age-associated alterations in humans. The method revealed also age-associated atrophy in cortical regions (cingulate, occipital, parietal), nucleus septalis, and the caudate. Manual measures performed in some of these regions were in good agreement with results from automatic measures. The templates generated in this study as well as the toolbox for SPM8 can be downloaded. These tools will be valuable for future evaluation of various treatments that are tested to modulate cerebral aging in lemurs.

## Introduction

Cerebral alterations associated with aging affect quality of life for millions of people. One of the most described alterations associated with aging is cerebral atrophy. Animal models of aging are important for providing a means of testing interventions. Most commonly, rodents are used but they do not display severe cerebral atrophy with aging, indeed non-transgenic rodents often show cerebral growth with age (Maheswaran et al., 2009). Animal models of disease leading to cerebral atrophy in humans, such as Alzheimer's disease do not recapitulate the loss of cerebral tissue seen in patients (Delatour et al., 2006; Maheswaran et al., 2009). Non-human primates do show age-related changes more closely resembling human aging (Andersen et al., 1999; Dhenain et al., 2000). One of these, the mouse lemur (*Microcebus murinus*), is used as a model of age-related neurodegenerative processes. This animal is small (12 cm, 60–100 g) with a short maximum lifespan of approximately 10 years in captivity. This primate can thus be a useful compromise between practicality and relatedness to humans for laboratory studies (Languille et al., 2012). Previous studies in this model have shown atrophy of distinct brain regions (cortical regions, hippocampus, the caudate nucleus, and white matter regions such as the splenium) (Picq et al., 2012) including atrophy of some hippocampal subfields (Bertrand et al., 2013) as well as increased levels of cerebrospinal fluid (CSF) (Kraska et al., 2011). Interestingly, in this non-human primate, cerebral atrophy has been shown to correlate with cognitive deficits (Picq et al., 2012). Currently this animal is used to evaluate the effect of various therapies against age associated alterations (Dal-Pan et al., 2011) or neurodegenerative diseases (Joseph-Mathurin et al., 2013). Previous studies of cerebral atrophy in mouse lemurs were based on the manual and tedious segmentation of selected regions of interest (ROI), or on the manual measurement of cortical thickness from T2-weighted magnetic resonance images (MRI) (Dhenain et al., 2003; Kraska et al., 2011; Picq et al., 2012). Although these studies had two reviewers for the delineation, reducing the inherent subjectivity of the technique, it is not practical to extend these techniques to large populations for routine assessment of morphological changes for more than a few regions as the workload becomes impractical. The same issue discourages manual studies of large cohorts of animals with various therapies or of longitudinal examination of developing pathology. In addition, manual studies are unable to uncover unknown regions of pathology, as such studies focus only on specific areas where changes are anticipated and not all anatomically distinct regions are identifiably distinct with usual image contrasts.

In human studies, morphometric assessment of structural change with age is generally performed with an automated procedure such as voxel-based morphometry (VBM) or the closely-related technique deformation-based morphometry (DBM). These methods can be used to detect changes with age on a voxel-by-voxel basis (Kalpouzos et al., 2009; Bergfield et al., 2010; Giorgio et al., 2010). Recent studies have shown that these methods can also be applied to animal brains and can be more sensitive than ROI-based methods (Lau et al., 2008; Sawiak et al., 2009; Ellegood et al., 2013). A principal advantage of the methods is that no region needs to be selected in advance for analysis and the whole brain can be assessed automatically in a user-independent manner.

In the present study, we apply VBM to the mouse lemur for the first time and identify specific areas of localized atrophy. In particular, atrophy was highlighted in some regions that cannot be manually outlined such as the nucleus basalis of Meynert, the nucleus septalis, the subiculum, the prepiriform cortex, Brodmann areas 13–16, the hypothalamus, the putamen, or the thalamus. In some cortical regions, cerebral atrophy was evaluated with both VBM and manual measures which have been previously validated. Strong agreement was found between the results obtained with the two protocols. The probabilistic gray matter (GM), white matter (WM) and CSF templates in addition to the population atlas images which were created during this study are made publicly available with this article in supplementary material so that other researchers using these animals in any context can readily apply VBM for morphological assessments.

## Materials and methods

### Animals and image acquisition

Thirty mouse lemurs aged from 1.9 to 11.3 years (7 “young” animals 2.2 ± 0.2 years, 11 “middle-aged” 4.8 ± 1.0 years and 12 “old” 8.3 ± 1.7 years, mean ± SD) were included in the study. All of the animals were born in a laboratory breeding colony (Brunoy, France Agreement number 962773) and all components of this study were in accordance with EU regulations relating to the use of animals in experiments. The research was conducted under the authorization number 91–326 from the “Direction Départementale des Services Vétérinaires de l'Essonne.”

The animals were scanned at 4.7 Tesla following a protocol previously described (Dhenain et al., 2003; Kraska et al., 2011). Briefly, they were anaesthetized with atropine (0.025 mg/kg subcutaneously) and isoflurane, their respiration rate and body temperature were monitored and controlled to ensure stability throughout the experiment. Images were acquired using an inversion-prepared three-dimensional fast spin-echo sequence with an isotropic resolution of 234 μm with T2-weighting (TR/TE_eff_/TI 2500/45/200 ms, ETL 16, NEX 1 and acquisition time 44 min). MR images were zerofilled to reach an apparent isotropic resolution of 117 μm.

### Automatic evaluation of cerebral atrophy with VBM

**Figure 1:**
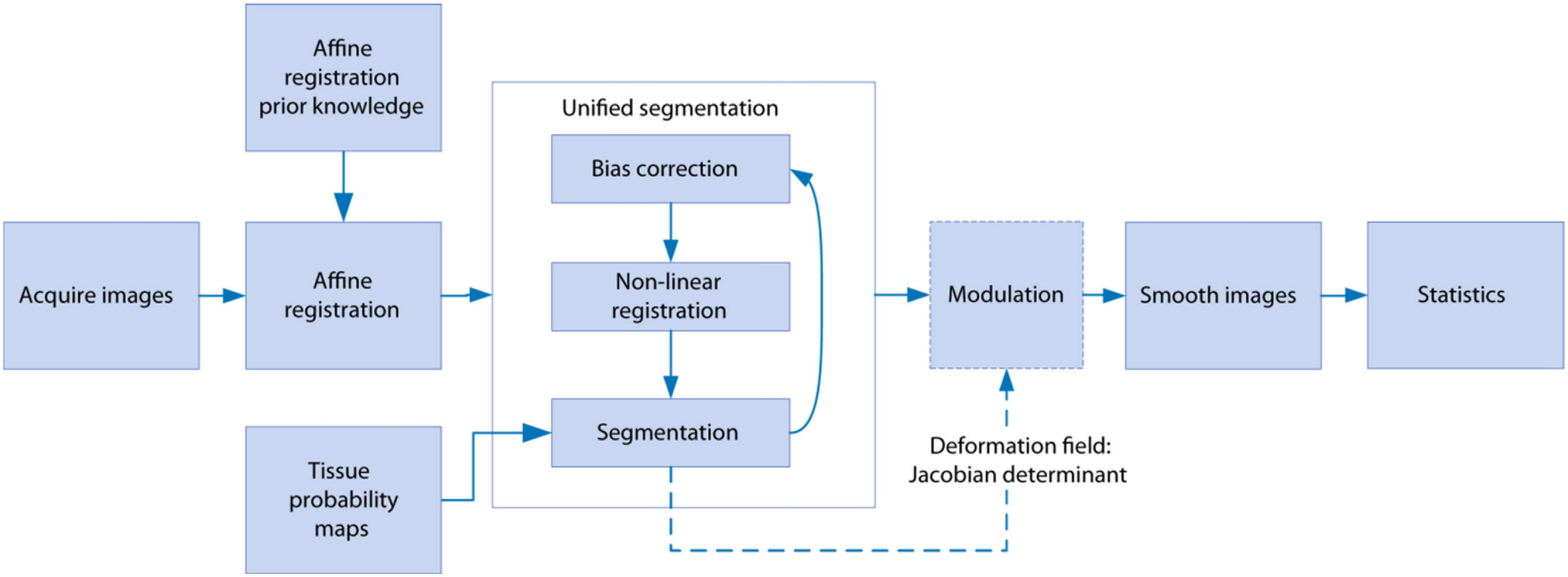
**The steps involved in the voxel-based morphometry study**. After image acquisition, affine registration adjusts the images to control for different head positions and scanner geometry as well as overall brain size. Unified segmentation iteratively warps the data whilst correcting for signal inhomogeneity due to the receiver coil and classifies each voxel as gray matter, white matter, or cerebrospinal fluid. The warping parameters are used to preserve the total amounts of each tissue in the modulation step, and smoothing serves the dual purpose of minimizing residual registration errors and reducing non-normality in the data for parametric testing in the statistics stage.

Image processing was performed using SPM8 (Wellcome Trust Institute of Neurology, University College London, UK, (www.fil.ion.ucl.ac.uk/spm) with the SPMMouse toolbox (http://spmmouse.org) for animal brain morphometry (Sawiak et al., 2013). An overview of the algorithm is given in Figure 1. Initial templates were constructed by using a single brain as a target for rigid registration to achieve a common alignment of each subject. The average image of the rigidly-aligned brains was segmented using a *k*-means algorithm (Mackay, 2003) with 4 segments: background, GM, WM, and CSF. These maps were manually edited to remove misclassified voxels, particularly around the edges of the brain where partial volume effects lead to mislabeling of GM voxels as WM. After smoothing with an isotropic Gaussian kernel of 600 μm, the maps for GM, WM and CSF were used with the unified segmentation algorithm of SPM8 (Ashburner and Friston, 2005). Affine regularization was set for an average-sized template, with a bias non-uniformity cut off FWHM of 10 mm, a 5 mm basis-function cut off and a sampling distance of 0.3 mm.

The resulting GM and WM portions were output in rigid template space and DARTEL (Ashburner, 2007) was used to create non-linearly registered maps for each subject and common templates for the cohort of animals. The warped GM portions for each subject were modulated with the Jacobian determinant from the DARTEL registration fields to preserve tissue amounts (“optimized VBM” Good et al., 2001) and smoothed with a Gaussian kernel of 600 μm to produce maps for analysis.

A general linear model was evaluated with the age of each subject with calculated total intracranial volume (TIV) as a covariate of no interest. Voxels with a modulated GM value below 0.2 were not considered for analysis and the sum of the tissue probability maps was used as an explicit mask. A one-tailed *t*-test contrast was set up to find areas where GM values decreased with age. A second model was fit to the WM images with the same parameters to investigate changes in WM and subcortical structures. To control for multiple comparisons an adjusted *p*-value was calculated to control the voxelwise false discovery rate (FDR) *q* < 0.01 (Genovese et al., 2002).

It is important to note throughout this study that the terms GM, WM and CSF refer to tissue classification in the MR images. In particular, some subcortical regions which are not strictly WM are labeled as such due to their appearance in the MR images. Throughout this article the classification of WM should be understood as including tissues with contrast similar to WM (e.g., white matter fibers or subcortical structures such as the thalamus).

### Manual evaluation of cerebral atrophy

**Figure 2:**
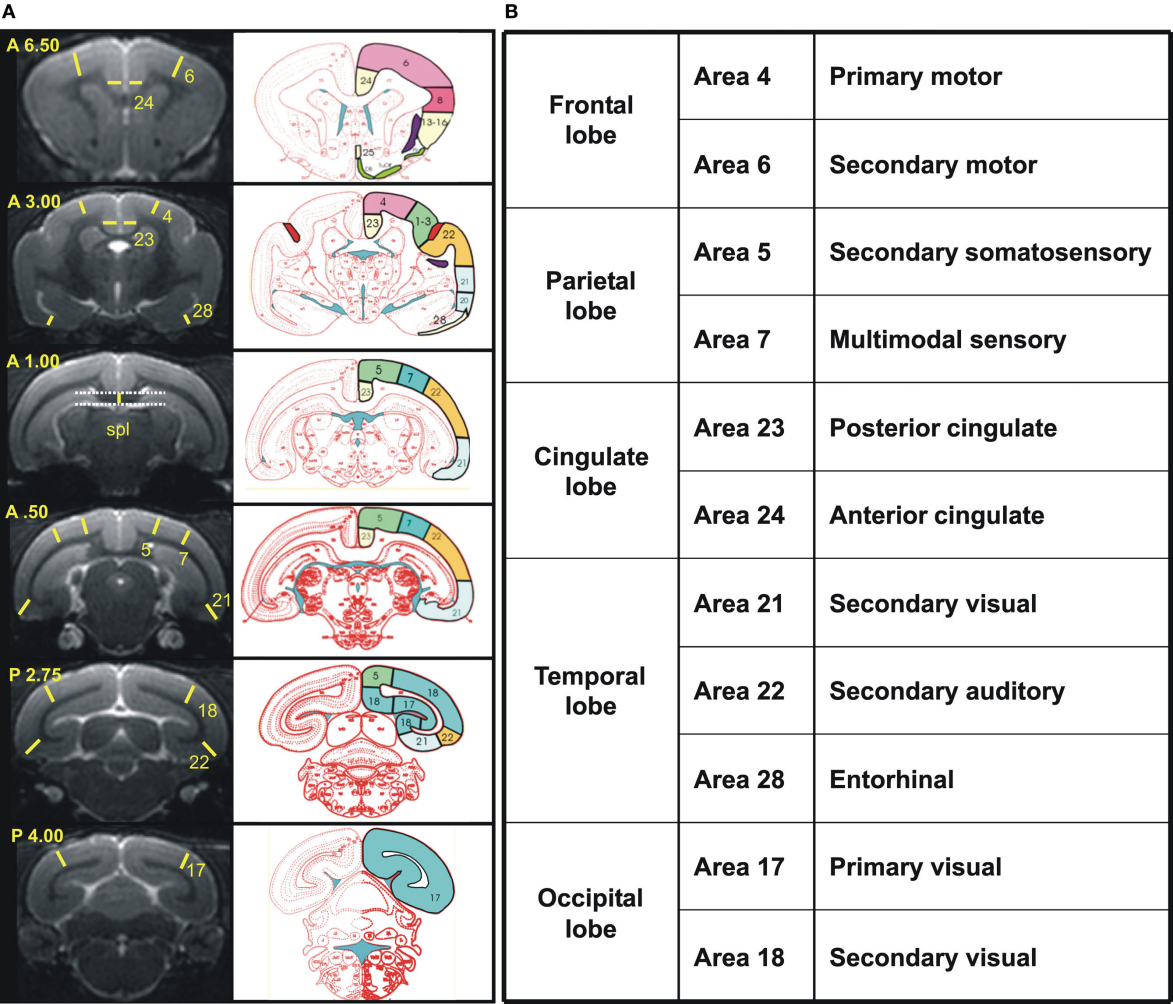
**(A)** Reference coronal slices used to measure cortex and splenium thickness in cortical regions. Left, Views of the six coronal MR images taken into account with yellow lines and numbers indicating the precise localization of each measure and the cortical area measured, respectively. Coordinates based on the stereotaxic brain atlas of Bons et al. (1998) are noted in the superior left corner of each slice. Right, schematic sections from the atlas of Bons et al. corresponding to the coronal slices on the left column with identification of the Brodmann's cortical areas measured (http://marc.dhenain.free.fr/Mouse-Lemur-Atlas/index-Mouse-Lemur-Atlas.html). Spl, splenium. **(B**) An overview of the cortical regions is shown in the table.

Cerebral atrophy was also evaluated on the basis of manual segmentations of the brains performed with BrainVISA and Anatomist freeware (http://brainvisa.info/) as well as ImageJ, according to previously established protocols (Picq et al., 2012). Briefly, first, before morphometric analysis, the brain images from the different mouse lemurs were rotated to be positioned in a similar orientation. Standard neuroanatomic landmarks were used to correct deviations in all three orthogonal planes: the sagittal plane cut through the middle of the interhemispheric fissure, and the horizontal plane was parallel to the superior border of the median part of the corpus callosum and rigorously perpendicular to the sagittal plane, in accordance with the stereotaxic brain atlas of the gray mouse lemur by Bons et al. (1998), which was used as a reference for all anatomical landmarks. The thickness of 11 different cortical areas was estimated based on five coronal slices corresponding to +6.5, +3.0, +0.5, −2.75, and −4.0 mm from the middle of the inter-ear distance (Bons et al., 1998). These regions could be grouped as the frontal, parietal, temporal, occipital, and cingulated cortices (Figure 2). The thickness of the splenium of the corpus callosum was measured for evaluating the age-related decline in white matter and was estimated from the coronal slice where it was the thickest. Given that fibers of the cingulum bundle and dorsal hippocampal commissure border on the splenium, the measure probably also included these two other structures.

The measures of cortical and splenium thickness were performed by a single experimenter. Intrarater reliability was assessed by measuring each region of interest (ROI) on 10 animals (three from the young group and seven from the older group) on two separate days at least 1 month apart and by calculation of intraclass correlation coefficients. All of the obtained coefficients ranged from 0.99 to 1. The mean measuring error was below 1% for all ROIs and the maximal measuring error under 2%. A second investigator measured a sample of 6 animals (two from the young group and four from the older group) in order to assess interrater reliability. All interrater reliability values were >0.90.

## Results

**Figure 3:**
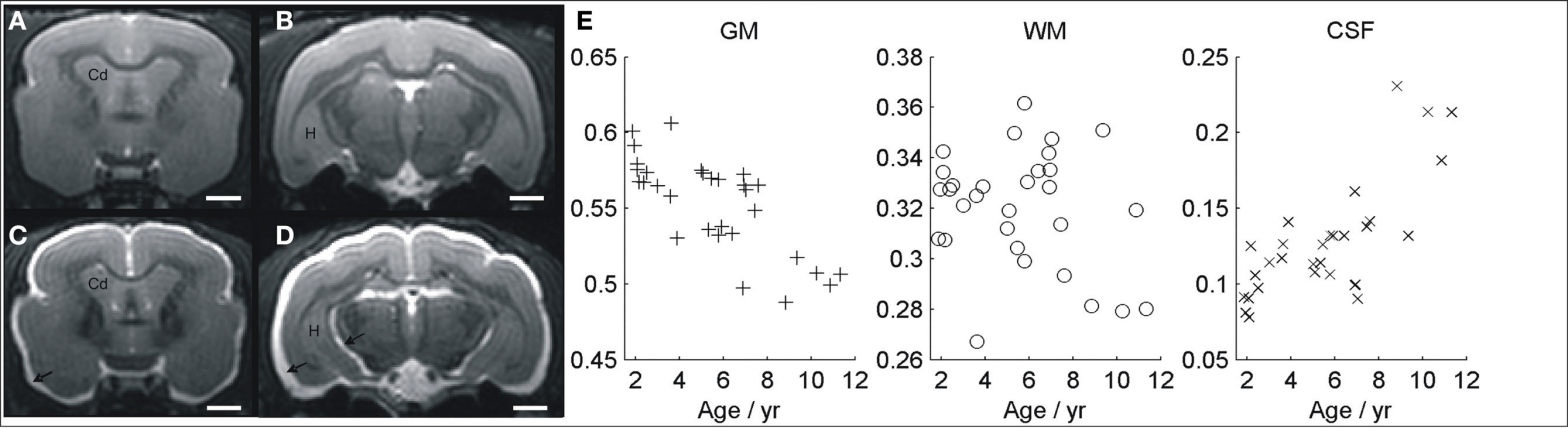
**MR image showing a young non-atrophied animal (A,B) compared with an example of old atrophied lemur (C,D)**. An increased cerebrospinal fluid associated to a severe atrophy process can be seen in the older animal (arrows). The evolutions of gray matter, white matter, and CSF volumes, evaluated with voxelwise studies, are shown in **(E)**. Cd, caudate nucleus; H, hippocampus. Scale bars = 2 mm.

Images of typical normal and atrophied animals are shown in Figures 3A–D. Gross cerebral atrophy leading to increased CSF around the brain can be clearly seen in some of the older animals. Evaluation of cerebral atrophy with VBM revealed a negative correlation between the total GM volume and age (*r* = −0.4, *p* = 0.02, Figure 3E). On the contrary, the total CSF showed a positive correlation with age (*r* = 0.8, *p* < 10^−6^, Figure 3E). No significant change was seen in regions classified as WM across the brain (*r* = 0.03, *p* = 0.4, Figure 3E).

**Figure 4:**
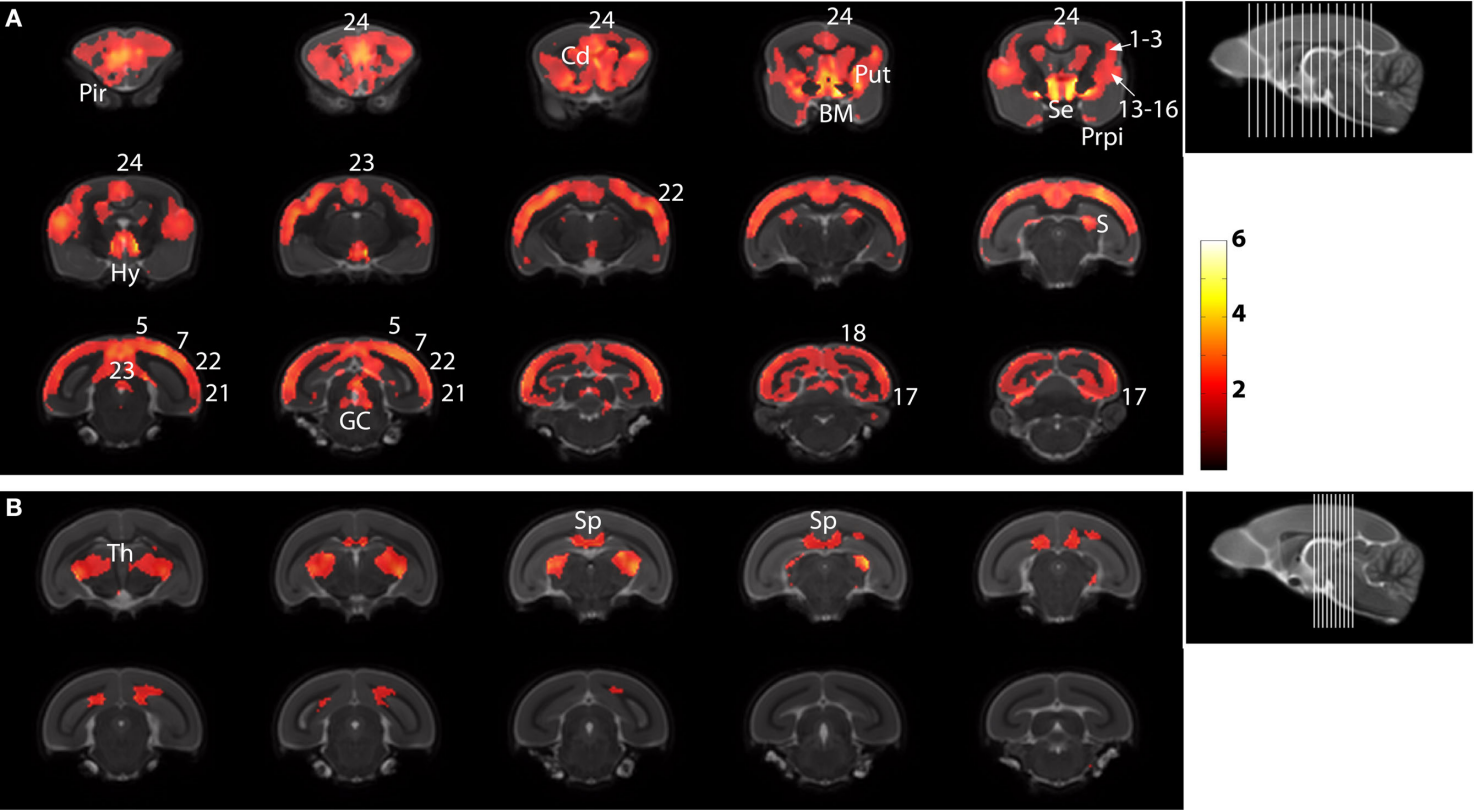
**Voxelwise areas showing decreasing GM values (A) and WM and associated subcortical regions values (B) with increasing age (*p* < 0.01 FDR-corrected)**. The labels represent the regions cited in Table 1.

**Figure 5:**
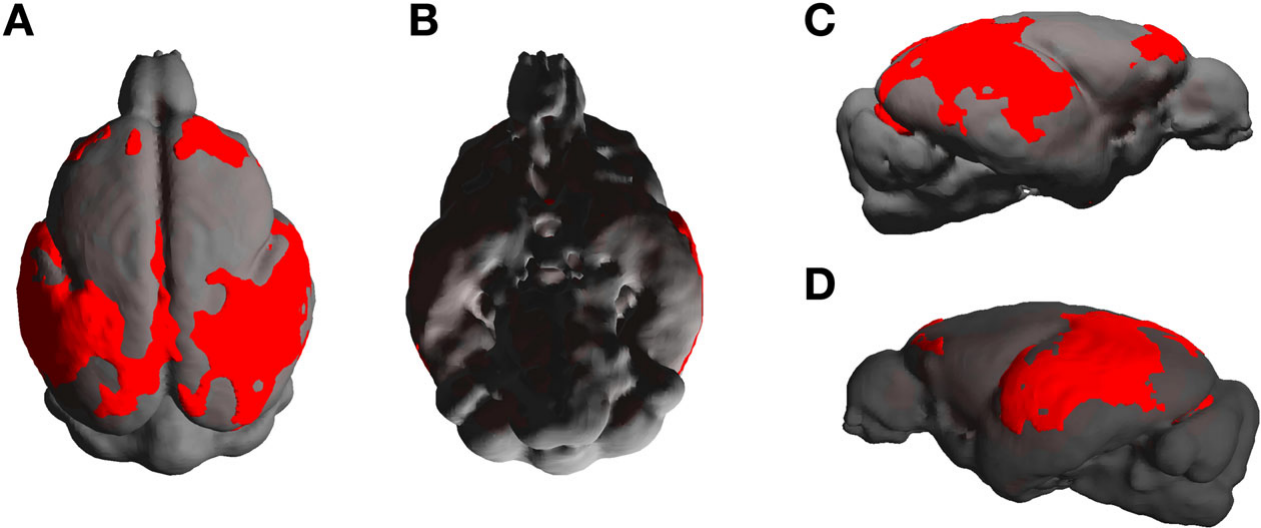
**External views [A: dorsal, B: ventral, C,D: lateral (right and left)] outlining age-associated cortical alterations in mouse lemurs**.

The statistical parametric map (SPM) for the VBM analysis of GM and regions classified as WM changes are shown in Figure 4. External views outlining cortical alterations are presented in the Figure 5. Also, full details of all of the clusters identified by VBM are shown in Table 1. Nearly all of the changes seen on SPM maps are symmetric with both sides of the brain affected. The majority of cortical regions displayed some atrophy with age, with the most prominent areas of reduction including the cingulate cortex, occipital cortex, parietal areas with frontal cortex partly spared. Subcortical regions such as the caudate or putamen were also particularly affected by aging. Other clusters such as the nucleus basalis of Meynert, the nucleus septalis, the subiculum, the prepiriform cortex, or the hypothalamus were also altered during aging. The maps corresponding to regions classified as WM show changes in the corpus callosum at the level of the splenium as well as a bilateral effect in the thalamus (Figure 4, Table 1).

**Table 1:** **Clusters showing significant atrophy on the basis of VBM analysis in mouse lemurs**.

**Name of the structure**	**Corrected *p*-value**	***t* score**	**Template coordinate—antero-posterior**	**Template coordinate—left-right**	**Template coordinate—dorso-ventral**	**Label in Figure 4**
Nucleus basalis (Meynert)	2.3E-07	11.37	−5.0	−2.3	−1.3	BM
Nucleus septalis	2.3E-07	11.33	−5.7	1.6	−1.3	Se
Hypothalamus	6.6E-07	9.92	−3.4	1.6	−1.3	Hy
Hypothalamus	1.5E-06	8.85	−4.6	−0.2	−1.6	Hy
Brodmann 7	3.2E-06	8.08	0.5	4.1	3.7	7
Nucleus septalis medialis	4.6E-06	7.83	−5.7	0.2	0.5	Se
Brodmann13–16 and 1–3	7.3E-06	7.50	−6.0	5.0	2.6	1–3
Putamen	8.4E-06	7.40	−5.7	3.0	1.0	Put
Anterior cingulate	1.2E-05	7.16	−8.3	0.2	2.4	24
Brodman22	1.3E-05	7.12	−2.1	6.0	2.6	22
Brodman13–16 and 1–3	1.4E-05	7.03	−1.6	−3.7	4.2	1–3
Brodmann22	1.5E-05	7.01	−2.1	−5.8	2.4	22
Subiculum	1.6E-05	6.93	0.2	2.7	1.4	S
Brodmann22	1.8E-05	6.82	−4.1	−5.8	1.2	22
Brodmann17	2.0E-05	6.72	5.5	−3.7	0.1	17
Brodmann18	2.5E-05	6.55	2.3	6.0	3.0	18
Brodmann17	2.5E-05	6.55	2.8	6.9	−0.6	17
Substantia grisea centralis	3.0E-05	6.39	1.6	0.2	1.0	GC
Brodmann17	3.0E-05	6.39	3.0	−6.7	0.5	17
Brodmann23	3.1E-05	6.36	0.7	0.0	3.5	23
Brodmann17	3.4E-05	6.30	6.0	3.4	0.3	17
Brodmann17	3.4E-05	6.28	4.2	6.0	1.9	17
Caudate	4.7E-05	6.04	−7.1	−1.6	2.8	Cd
Brodmann18	4.8E-05	6.02	3.9	−1.4	1.7	18
Olfactory bulb	5.5E-05	5.92	−11.0	0.7	0.5	OB
Brodmann22	6.8E-05	5.75	−0.9	7.3	0.7	22
Caudate	7.3E-05	5.70	−6.9	2.3	3.0	Cd
Brodmann22	7.7E-05	5.66	−1.1	−6.9	0.5	22
Brodmann22	9.4E-05	5.52	0.7	−6.2	1.4	22
Piriformis cortex	1.1E-04	5.42	−8.3	−1.4	−0.6	OB
Brodmann20	1.1E-03	4.00	−2.1	−6.2	−2.5	20
Brodmann20	1.8E-03	3.75	−1.6	6.7	−2.2	20
Regio Praepiriformis	2.4E-03	3.60	−5.0	2.7	−3.9	Prpi
Thalamus	3.1E-08	5.66	0.20	4.35	0.52	Th
Thalamus	8.2E-06	5.26	2.15	−0.48	0.98	Th
Splenium of the corpus callosum	9.2E-03	5.58	0.85	0.67	2.36	Sp

*Spatial coordinates make reference to the template that is provided as a complementary data with this article. On this template, zero in the antero-posterior direction corresponds to zero in the Bons atlas (Bons et al., 1998); zero in the right-left direction correspond to the interhemispheric region; zero in the dorso-ventral direction corresponds to the anterior commissure*.

Using the deformations required to bring each subject into common template space, it is possible to invert with exaggeration to make “caricatured” young and old brains to highlight visually the effects seen. We used the DARTEL deformations to create such images with gradually progressive factors to create movies of caricatured young to caricatured old brains. These videos, included as supplementary materials, highlight the differences seen in Figure 4 in a dynamic fashion.

The evaluation of cortical atrophy on the basis of manual segmentation revealed a linear correlation between cortical thickness and the age of the animals in 7 out of the 11 studied regions. The atrophied regions were the cingular cortices [Brodmann areas 23 and 24 (*r* = −0.67 and −0.66, respectively, *p* < 0.0001; most atrophied regions), the parietal cortices (areas 5 and 7, *r* = −0.41, *p* = 0.01 and *r* = −0.44, *p* = 0.008, respectively), the lateral temporal cortices (areas 21 and 22, *r* = −0.41, *p* = 0.01 and *r* = 0.49, *p* = 0.003, respectively) as well as the occipital cortex (area 18, *r* = −0.48, *p* = 0.003)]. All of these regions were also detected as atrophied when we used the VBM-based analysis. The thickness of area 17 in the occipital region was almost significantly correlated with the age of the animals (*p* = 0.1) and this region was found to be atrophied by VBM. The three last cortical regions that were not atrophied with aging were the frontal regions (areas 4 and 6, *r* = −0.06, *p* = 0.039 and *r* = 0.07, *p* = 0.035, respectively) and the entorhinal cortex (area 28, *r* = 0.17, *p* = 0.019). These areas were not detected as atrophied when we performed the VBM analysis. Manual evaluation also revealed an inverse correlation between the thickness of the splenium and the age of the lemurs (*r* = −0.6, *p* = 0.0003). This region was also found to be atrophied when we used the VBM method. These findings together show that VBM is in good agreement with measures of the cortical or splenium atrophy made manually.

Finally a good correlation was detected between the total CSF volume and the atrophy of the cingulated regions (23 and 24), the temporal regions (22 and 28), the frontal areas 4 and 6, and the splenium (all *ps* < 0.05), which suggests that the global CSF increase detected by SPM was originating from various brain regions. Similarly the global GM atrophy detected by SPM was also correlated to the atrophy level of various cortical regions such as the cingulated cortex at the level of the area 23, the parietal region (area 7), the temporal region (area 21), and the occipital regions (areas 17 and 18) (all *p* < 0.05), and thus originating from various cortical areas.

## Discussion

In this study, we evaluated cerebral atrophy in the mouse lemur model of cerebral aging by using two complementary methods based on voxelwise comparison and manual segmentation.

The voxelwise approach revealed for the first time cerebral atrophy in regions that cannot be outlined with manual methods. For example, we detected atrophy of the nucleus basalis of Meynert, subiculum, prepiriform cortex, Brodmann areas 13–16, hypothalamus, putamen, and the thalamus. The voxelwise approach also revealed atrophy in the nucleus septalis. The voxelwise and manual delineation of tissues also highlighted age associated atrophy in some regions such as the cingular, parietal, temporal, and occipital cortices and the splenium as well as a preservation of some regions such as the frontal and entorhinal cortices.

Most of the brain regions detected as atrophied for the first time in mouse lemurs have also been reported to be vulnerable to aging in humans. These include the nucleus basalis of Meynert (Lowes-Hummel et al., 1989), prefrontal cortex (Raz et al., 1997), cingulate cortex (Kalpouzos et al., 2009), the subiculum (La Joie et al., 2010), the thalamus (Sullivan et al., 2004) and the putamen (Raz et al., 2003). Thus the pattern of age-related brain degeneration in mouse lemurs is very close to that described in humans. Some of these atrophied regions are of particular interest. For example, the nucleus basalis of Meynert is involved in cholinergic transmission, a neurotransmitter system that is impaired early in the aging process (Bartus et al., 1982) and that was shown to be altered in aged lemurs (Mestre and Bons, 1993; Dournaud et al., 1994). The Brodmann areas 13–16 and cingulate cortex are involved in executive function in the lemurs and can be viewed as equivalents of prefrontal regions (Le Gros Clark, 1931). Impairments in executive functions are a common sign of cognitive decline in the elderly (Gabrieli, 1996). Previous publications demonstrated that executive functions are also especially vulnerable to aging in mouse lemurs (Picq, 2007; Picq et al., 2012) and revealed a relationship between alterations of executive functions and the atrophy of the nucleus septalis (Picq et al., 2012). Our report suggests that the areas 13–16, together with cingulate areas, are also candidate regions to be examined for determining the neuroanatomical correlate of the age-related executive decline in mouse lemurs.

In previous studies, we highlighted cerebral atrophy in mouse lemurs thanks to manual delineation of regions of interest. Most results from these manual studies are consistent with our VBM study (Picq et al., 2012). One partial discrepancy between the current and previous studies concerns the entorhinal cortex. In our previous cohort (Picq et al., 2012), the entorhinal cortex was atrophied whereas the reverse was found in the current study. However, in our previous cohort, only a limited subpopulation of older mouse lemurs underwent entorhinal atrophy. In the present study, four animals including three old and one middle-aged lemurs, had an atrophy level of the entorhinal cortex that was twice the standard deviation of the young animals, suggesting pathological atrophy. Hence, it is possible that the only difference between the two studies was the proportion of aged animals affected by this supposed pathological atrophy.

Few studies to date have directly compared voxelwise approaches to manual segmentation in animals, though it has been shown that VBM is more sensitive to subtle differences than manual volumetry in mice (Sawiak et al., 2009). Here, 12 regions were analyzed manually and there was only one small discrepancy between the voxelwise and manual methods. It concerned the occipital area 17 that was detected as atrophied with the voxelwise approach while only a trend toward atrophy was detected with the manual method. This small discrepancy can be explained by a higher sensitivity of the VBM method or because it assessed the whole cortex while the manual method involved only one measure of the cortex at a single location. The strong similarity between results obtained with voxelwise and manual approaches thus reinforces the validation of the voxelwise approach in lemurs.

The voxelwise approach will thus allow operator independent measures of cerebral atrophy in large cohort of mouse lemurs by evaluating the whole brain without any a priori hypothesis. The method is fast as compared to manual segmentations and may be more sensitive as suggested by its ability to detect atrophy in the area 17 while only a trend toward atrophy was seen in this region with the manual method. In order to facilitate the use of voxelwise studies in animals, our group designed a toolbox that can be used with SPM8 and made this toolbox freely available for the scientific community. This toolbox allows to manipulate easily animal images within SPM8 that is designed for human studies. One of the remaining difficulties when dealing with animal studies is to define an initial template that can be used as a starting point for VBM studies. Here, we created mouse lemur templates and provided these templates as Supplementary Material, in order to facilitate further studies by the scientific community interested by this small primate model.

Although the voxelwise technique allows to characterize the cerebral atrophy in aged lemurs, it is important to note some potential disadvantages of the method. In general the VBM processing pipeline is more sophisticated than that of ROI volumetry with the possibility at each stage of a systematic bias being introduced between groups that will cause subsequent rejection of the null hypothesis. It is important therefore when interpreting VBM results to eliminate artificial causes for differences between processed images that do not originate from genuine biological differences. In addition, image artefacts and voxel-misclassification is not as problematic with regional techniques as typically a large structure is integrated and under expert review artefacts are more readily identified and treated appropriately. Change in tissue structure that are not shape changes (especially, for example, iron deposition) will at least to some extent be sequence dependent and can cause the classification of affected voxels to change (Abbott et al., 2012). For this reason, it is important not to neglect this possibility when interpreting VBM findings and not to assume automatically that differences must be volume changes. In addition, it is worth highlighting past controversies regarding VBM applied to imperfectly registered images (Bookstein, 2001). The issue is that if the voxels compared are not truly homologous between individuals then a voxelwise comparison is difficult to interpret, particularly with the idea that with perfect registration there should be no differences left between images to detect. Generally, a consensus has emerged that by smoothing the data and taking account of tissue gained or lost in the registration process by modulating the tissue maps with the Jacobian determinant (as performed here) that the process is valid (Ashburner, 2001).

Finally, voxelwise techniques based on group comparisons do not detect individual cases of cerebral atrophy but rather those differences that are consistent across animals. The detection of atrophy in individual cases can be useful to include or exclude some animals in preclinical drug trials for example. Thus voxelwise techniques should not be used if the aim of the study is to detect individual animals with a particular atrophy pattern. This is highlighted by the apparently spared hippocampal regions in Figure 4. Previous work has shown that the temporal pole displays atrophy in these animals, but it is particularly heterogeneous. For voxels to be significant, the differences seen have to be large in comparison with the variability of the population. Larger studies will be required to detect changes in heterogeneous populations and indeed automated techniques such as VBM as validated here will be required to conduct these studies.

## Conclusions

As a conclusion we have demonstrated that VBM can be used in mouse lemurs to directly identify age-associated atrophied regions with more precision and sensitivity than previous methods using region-based analysis. In particular, some brain regions such as the nucleus basalis of Meynert, subiculum, prepiriform cortex, Brodmann areas 13–16, hypothalamus, putamen, or thalamus were shown, for the first time, to be vulnerable to aging in lemurs. Our technique can be readily adapted to longitudinal studies which will be more sensitive to age-related changes. The templates generated in this study can be downloaded as well as the toolbox for SPM8 used for the analysis here. These tools will be valuable for future evaluations of the effects of various treatments that are tested to modulate cerebral aging and age-associated pathology in lemurs.

### Conflict of interest statement

The authors declare that the research was conducted in the absence of any commercial or financial relationships that could be construed as a potential conflict of interest.
